# Embedding Clinical Reasoning into an Undergraduate Medical Curriculum: A Multi-Stakeholder Perspective

**DOI:** 10.5334/pme.2448

**Published:** 2026-03-25

**Authors:** Martine Chamberland, Isabelle Boulais, Jean Setrakian, Linda Bergeron, Tim Dubé

**Affiliations:** 1 Department of Medicine, Université de Sherbrooke, Sherbrooke, Quebec, Canada; 2 Université de Sherbrooke, Sherbrooke, Quebec, Canada; 3 Department of Family Medicine and Emergency Medicine, Université de Sherbrooke, Sherbrooke, Quebec, Canada

## Abstract

**Introduction:**

Medical educators agree on the importance of teaching clinical reasoning (CR) at the undergraduate level with a deliberate curricular approach. Despite recent recommendations on how to reach this objective, there are limited empirical data on how it unfolds once implemented. This study advanced the understanding of how a deliberately designed CR curriculum was experienced and contributed to students’ CR development. Our study sought to add to the conversation on undergraduate CR curricula by providing data that could be relevant and applicable across other learning contexts.

**Methods:**

We conducted a qualitative descriptive study in the context of a competency-based medical program that is interdisciplinary, with spiral integration. Through nine focus groups, we sought the experience of curriculum designers, teachers, and students on how the CR curriculum embedded into the program contributes to students’ CR development. Data were analyzed using a thematic approach.

**Results:**

We identified four overarching themes: 1- Global coherence of the curriculum around the development of CR; 2- Interdisciplinary integration to enhance CR development; 3-Cognitive load of students and pervasive time constraints; 4- Active teaching methods that promote the development of CR.

**Discussion:**

From the different stakeholders’ perspectives, students acquired various knowledge types in CR domains and applied them to problem-solving. Stakeholders’ experiences with the implemented CR curriculum matched the initial intent but presented some challenges as well. These findings underscored both the feasibility and the complexity of embedding CR within an undergraduate medical curriculum. Careful attention is required to support students and teachers.

## Introduction

Clinical reasoning (CR) is at the heart of medical expertise. Medical educators increasingly agree on the need to teach CR and its core components at the undergraduate level, with a curricular approach that is explicit, intentional and spans the entire course of undergraduate training [1,2]. Although general recommendations for designing such a curriculum and some descriptions of CR curricula have been recently published, there is still limited empirical evidence on how it unfolds in real-world settings once implemented.

Two scoping reviews [3,4] have highlighted the scarcity of detailed clinical reasoning curricula at the preclinical level. The UK Clinical Reasoning in Medical Education (CReME) Consensus statement group addressed this gap in 2021 by publishing proposals based on iterative discussion, agreed ideas, and the literature review on ‘what’ to teach and ‘how’ to teach CR in a curriculum [1]. The CReME group identified five domains of CR: (1) CR concepts, (2) History and physical examination, (3) Choosing and interpreting diagnostic tests, (4) Problem identification and management, (5) Shared decision-making. Each of these domains requires specific knowledge, skills and behaviours. They proposed a series of evidence-informed teaching strategies aiming at building knowledge and understanding, such as self-explanation, structured reflection, practice with feedback, structuring knowledge around problem-specific concepts and retrieval practice. They also proposed that educators design and implement a formal CR curriculum that is longitudinal, adapt strategies to the learner’s level, and is fully integrated into the program [1].

Since this consensus statement, a few descriptions of CR curricula, translating these proposals into practice, have been published [5,6,7,8,9]. The DID-ACT (Developing, implementing and disseminating an adaptive CR curriculum for healthcare students and educators) consortium group envisioned, designed, and piloted a generic CR curriculum of 25 learning units and a ‘train-the-trainer’ program that could be ‘imported’ and adapted by HPE programs [6,7]. Singh et al. detailed the development process and the implementation of a CR curriculum within a 5-year program (University of Manchester, UK) [5]. To implement it in an existing program, teaching materials and assessments were reframed to highlight CR. Mallory et al. described a theory-driven introductory course targeting clinical diagnosis and spanning over 15 months in the first two years of the undergraduate medical program [9]. Rowat and Suneja reported the development and implementation of a CR curriculum concurrent with a whole new program, articulated around three longitudinal strands (Mechanisms of health and disease, Medicine and society, Professional skills) and fully integrated within the program [8]. In comparison with the previous cohort, the first cohort of students entering the later curriculum demonstrated at 18 months a significant increase in total score on the Diagnosis Thinking Inventory (DTI) test. Among these reported CR curricula, only the one described by Rowat and Suneja [8] included some post-implementation outcome data.

There remains a lack of empirical research examining how such curricula are experienced in practice by learners and faculty. This is important because designing and implementing a CR curriculum is a highly complex and challenging educational endeavour. Understanding the perspectives of those experiencing the curriculum is essential to determine whether what was intended is realized in practice. Furthermore, such perspectives may contribute to a more nuanced understanding of clinical reasoning curricula and help identify overlooked aspects, barriers, and facilitators that are relevant across educational contexts, thereby informing future curriculum development and implementation efforts.

The present study explores stakeholders’ perspectives on how a preclinical CR curriculum contributes to students’ CR development.

## Methods

### Study design

We conducted a qualitative descriptive [10] study using focus groups [11] to delve into stakeholders’ different perspectives on how the intended preclinical CR curriculum was experienced and how it contributed to students’ CR development. This methodology was selected to describe participants’ views and experiences without imposing a priori theoretical interpretations. This research project received approval from the institutional review board (#2022-3650). All participants gave their consent to participate in this study.

To provide a context for interpreting our findings, we first describe the educational setting in which the study was conducted. Although the curriculum was implemented prior to the publication of the recommendations by Cooper et al. [1], we previously conducted a document analysis to inform the present study and observed a strong alignment between the CR curriculum and the CReME recommendations. This alignment provided the relevance for examining how this specific CR curriculum is experienced by stakeholders in practice. Moreover, since the program was in its sixth year of implementation, this experience could help stakeholders share their perspective.

### Context of the study

A renewal of the four-year undergraduate medical program took place in 2017 at a francophone university in Canada (Université de Sherbrooke in Québec). The program, on four sites across a vast geographical distribution, with about 210 students per cohort, entails two and a half years of preclinical studies and 18 months of clerkship. The curricular approach can be described as competency-based, problem-oriented, longitudinal, interdisciplinary, with spiral integration.

From the very first year and throughout the program, CR is explicitly taught and embedded within the teaching of basic and clinical sciences from diagnosis to management, history-taking, physical examination, communication skills, collaboration and professionalism, highlighting the pivotal role of patients as partners in the entire clinical process. The design is based on a CR definition similar to Daniel’s [12]: “A skill, process, or outcome wherein clinicians observe, collect and interpret data to diagnose and treat patients. Clinical reasoning entails both conscious and unconscious cognitive operations interacting with contextual factors such as the patient’s unique circumstances and preferences and the characteristics of the practice environment” (p. 902). The following theoretical frameworks constitute the foundation of the development of clinical reasoning in the curriculum: theory of medical expertise (knowledge development and organization and illness scripts [13]), and dual process theory [14]. The teaching strategies listed by Cooper et al. [1] are operationalized through a variety of methods deliberately sequenced and all organized around clinical problems of increasing complexity over the course of training. The first two years are structured into 23 learning units (LU), each lasting four to six weeks and centred on two to three undifferentiated clinical problems. Ten integration weeks (IW) are interspersed throughout the first two years to revisit and consolidate the content covered. Each LU-IW exemplifies the fully integrated preclinical educational process aimed to support CR development and will be the specific context of the present study described below.

#### Description of a Learning Unit and Integration Week

**Figure 1:**
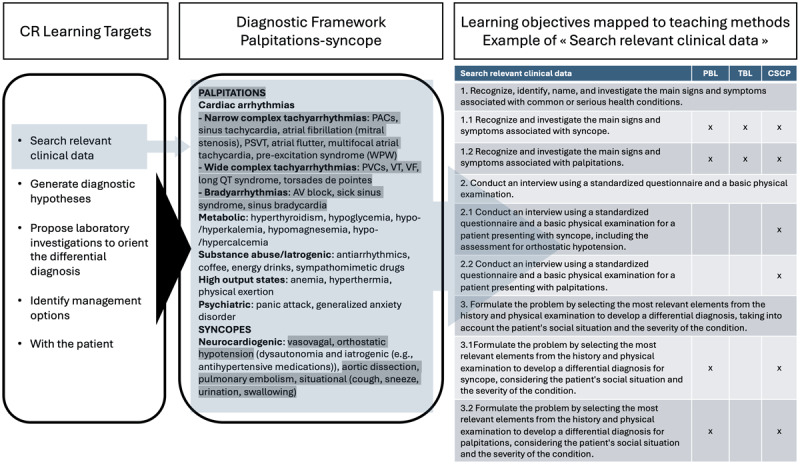
CR learning targets linked to a diagnostic framework for palpitations and syncope. The example illustrates specific learning objectives for “searching relevant clinical data”, each mapped to teaching methods designed to support achievement of objectives. PBL = Problem-based learning; TBL = Team-based learning; CSCP = Clinical Skills Communication and Professionalism.

To better illustrate the educational design, we will describe a specific LU of the second year: the Thoracic syndromes LU. During this LU, students go through the content related to three clinical problems (1- Palpitations and Syncope; 2- Chest Pain; and 3- Dyspnea, Cough, and Bronchospasm) while pursuing learning objectives directly and explicitly linked to CR domains as well as to specific diagnostic entities. For each problem, the content is systematically organized within a general diagnostic framework, provided to students as an outline, which highlights the diagnostic entities addressed during the LU. Then, for each clinical problem and for each CR target, specific learning objectives delineate the knowledge to be acquired by students (e.g., basic sciences, clinical sciences, history and physical examination, laboratory, pharmacology, communication) to enable students to reason through the problem and determine a general management plan. These specific objectives are distributed across the different teaching methods (e.g., Problem-based learning [PBL]; Team-based learning [TBL]; Clinical Skills Communication and Professionalism [CSCP], workshops: see Supplementary file 1) through deliberate, explicit content mapping. Figure 1 illustrates this organization with a sample for one problem (Palpitations-Syncope) and one CR target that is “Search relevant clinical data”.

Purposeful oriented data collection is taught with interviewing and physical examination skills. It is linked to the problem and the specific diseases studied during the LU. Accurate problem representation is emphasized and coached repeatedly. Diagnostic frameworks relevant to the problems are explicitly put forward at the very beginning of the LU and are progressively constructed by students. Even at this early phase in the program, diagnostic tests and their interpretation, the bases for management, and patient-centeredness are emphasized. Some aspects of CR domains were not clearly identified in the LU studied: medical/cognitive errors, evidence-based physical examination, and reflective practice of CR. These aspects are better addressed at a later stage of students’ CR development.

During the IW, no new knowledge or skills are to be learned. Instead, the goal is for students to review and deepen knowledge and skills acquired in the two previous LU, and apply these to new clinical cases, either more complex or in a more authentic context. To achieve this, two new teaching methods are used, self-explanation and structured reflection activity [15,16] and small group standardized patient (SP) sessions.

### Participant recruitment

Designers, teachers of the Thoracic syndromes LU, teachers of the related IW, and students were the targeted participants for this study. We selected the mid-second year “Thoracic syndromes” LU and the following IW to conduct the study with second-year students who had sufficient exposure to the recurring educational design to provide a well-informed perspective. Designers were a multidisciplinary group of educators, including undergraduate medical faculty and education specialists with expertise in student-centered learning and competency-based curriculum design. They designed and mapped the curriculum undergraduate competencies and developed supporting materials for faculty and students using local expertise. Approximately 50 teachers, distributed across the four campuses, are involved in the activities of the LU, while approximately 45 teachers, distributed across campuses, are responsible for the activities of the IW.

The principal investigator sent emails to each group of participants. The designers of the first two years of the curriculum were identified by the team, and all were invited to participate in the study. The program provided the names and emails of teachers of the LU, teachers of the IW, and students. Participants were recruited across the different campus sites.

### Data collection

The data collection took place in January and February 2023, immediately after the “Thoracic syndromes” LU and its following IW. Focus groups were purposely scheduled immediately after the LU-IW to harvest recent experiences and fresh impressions. A tailored interview guide was developed for each target group, structured around teaching methods in place, content taught, and the sequence of the LU and IW that supported the development of students’ CR (see Supplementary file 2). A research coordinator (LB) with extensive experience in this area led online focus groups of approximately one hour, using Microsoft Teams, for students and teachers, and in-person for designers. She met each stakeholder group separately to ensure homogeneity within the discussions. The research coordinator mainly asked the targeted questions and allowed the participants to interact among themselves, building on each other’s comments.

### Data analysis

We conducted an inductive thematic analysis based on Braun and Clarke’s [17] approach. Consistent with a qualitative descriptive approach, we stayed close to the data and used participants’ language to describe how the curriculum was experienced. The verbatim of the different stakeholders’ groups was first analyzed separately. In MS Word, LB reviewed all interviews and conducted initial coding, which MC then refined. Then all excerpts and corresponding codes were transferred to Excel. LB grouped the codes into preliminary themes and reviewed them collaboratively with MC. Themes were generated inductively from the data to address our research question regarding how the preclinical CR curriculum was experienced and how it contributed to students’ CR development from the perspective of the different stakeholders. These themes, supported by verbatim excerpts, were discussed with the research team to identify potential gaps. Final themes were then established by LB and MC, integrating insights from all stakeholder groups into a coherent framework.

### Reflexivity statement

The team included three internal medicine physicians (JS, MC, IB) with extensive experience in teaching and research, two (MC, IB) of whom are trained in medical education and well-acquainted with the program and its instructional methods, as well as one researcher (TD) and one research coordinator (LB). Throughout the study, we engaged as a group in periodic discussions. We kept a logbook of meeting reports and decisions made, as well as new thoughts arising during our data analysis that could later be shared with the team to ensure a nuanced and balanced interpretation of the data.

## Results

Nine focus groups with the different stakeholders gathered their field-based perspective about students’ CR development within a LU-IW (see Table 1).

**Table 1:** Number of groups and participants by stakeholders.


GROUPS	n OF GROUPS; n OF PARTICIPANTS

Curriculum designers	1 group; 5

Teachers involved in the LU	3 groups; 5, 3, 2

Teachers involved in the IW	2 groups; 3, 2

Medical students	3 groups; 5, 3, 2


The following section presents four overarching themes identified in the focus group discussions from the perspectives of designers, students, and teachers: 1) Global coherence of the curriculum around the development of CR; 2)Interdisciplinary integration to enhance CR development; 3) Cognitive load of students and constant time constraints; 4) Active teaching methods that promote the development of CR. The first three themes are closely tied to how the overall organization of the CR curriculum was perceived and experienced, and the last one is related to the teaching strategies and methods.

A first theme was ***Global coherence of the curriculum around the development of CR***. Clinical presentations provide the curriculum’s structural framework and inform the organization of each LU and IW. Participants perceived that clinical problems form the basis of the curriculum design (“Everything is based on clinical cases” [Designer2]), and the sequence of the clinical cases is meticulously planned according to the knowledge to be acquired, progressing in complexity and realism. According to designers and teachers, this progression enables a gradual and relevant enrichment of knowledge that is immediately applicable to CR. “It really allows us to add new layers of paint. And since it comes back again, you can really feel it being reactivated. I don’t have the kind of memory that retains things unless they’re anchored somehow — either with a patient or with something concrete. So I find that really helpful in itself.” (Student3–3)

Designers and students noted that the use of clinical vignettes facilitated understanding of basic sciences within their context, provided explanations for clinical elements, enhanced retention, and allowed the direct application of basic sciences in diagnosis and management.

A second theme, ***Interdisciplinary integration to enhance CR development***, focused on the interdisciplinary integration that promotes CR development through cases designed to reflect real-world practice. This, in turn, supports the organization of knowledge, the integration of diagnostic alternatives from different systems, and a broad, practice-relevant approach to clinical problems. The following teacher’s quote illustrated this strength. “Our students are much more open to differential diagnosis, because chest pain isn’t just about the heart, so they’re much quicker to think of all the possible diagnoses and then explore elsewhere”. (TeacherLU2–5).

Despite the strength of interdisciplinary integration, there were some challenges. Designers and teachers acknowledged that interdisciplinary integration requires careful preparation for teachers who may sometimes feel outside of their comfort zones: “I handle the thorax, but I cover a part of cardiology, and a part of pulmonology; I don’t need to tell you that I am more comfortable with the pulmonology part than the cardiology part.” (TeacherLU1–2) Therefore, training for teachers and high-quality teaching materials to support them are required.

Interdisciplinary integration involves the segmentation of each system content (e.g. respiratory system) over time, which may challenge teachers and students at some point. For teachers, this approach tended to fragment their teaching interventions over the different LUs, thereby complicating the provision of cohesive educational support to students.

From the perspectives of both students and teachers, the segmentation and temporal distribution of content sometimes left students feeling they had not explored topics in sufficient depth, and this could limit the retention and integration of knowledge: “So I find that sometimes, by trying to tell us “we will revisit the material multiple times”, we don’t go deep enough for my way of understanding things. And that leaves me in a fog.” (Student1–2)

A third theme was the ***Cognitive load of students and constant time constraints***. Three subthemes under this theme were related to the general organization. The first subtheme refers to how understanding the educational organization of a LU and mastering the tools and methods contribute to the students’ cognitive load. The complexity of the organization of each LU-IW required initial, explicit, and repeated guidance for students to understand and adopt it. The following quote from a student illustrated this point: “The first time I opened the objectives, it was quite a shock. It seems complex, a bit daunting. And you don’t really know how to navigate it at first. (Student3–4).” The program provides quality training and materials (student notebook, objectives, syllabus) to support students in their learning tasks. Despite increasing familiarity with the curriculum across the LUs, some students reported an ongoing need for repeated clarification of the program’s curricular organization. To reduce cognitive load, students have developed strategies for sharing notes and reading materials.

The second subtheme highlighted the fact that, despite the benefits of integrating CR along with knowledge building, the volume and intense pace of learning added to the students’ cognitive load. The integrated curriculum requires students to acquire a dense amount of knowledge at a rapid pace and learn how to use this knowledge gradually to develop CR. This sequence of acquisition, integration, and utilization of knowledge is cognitively demanding for students and requires time, which is scarce in the program structure. Furthermore, some students expressed difficulty in grasping the vast amount of material needed to prepare for educational activities. The workload is heavy and the references complex. “But I think overall, I’d like there to be less material, to feel less like I’m cramming during units, and have more time for self-directed learning.” (Student1–2) Teachers reported that some students experience anxiety when navigating and rapidly integrating the curriculum content.

The third subtheme pointed to how the time pressure created by balancing IW activities with preparation for the subsequent week’s assessments adds to students’ cognitive load.

A fourth theme, ***Active teaching methods that promote the development of CR***, focused specifically on teaching strategies and methods. Active and student-centred teaching methods support knowledge building from basic to clinical sciences and are sequenced to allow students to combine knowledge, apply it and practice CR repeatedly.

“It’s good to start with Problem-Based Learning because it focuses on a single, common problem, so we understand it well. Then, in […] the Team-Based Learning activity, it’s like several small problems, seen in succession, which helps us in our differential diagnosis to see more. I really like that we finish with a clinical skills session where we can really apply everything we’ve learned during the week. Often, teachers will make connections with their clinical practice.” (Student3–2)

“All the teaching methods always come back to clinical vignettes that force the student to analyze. So, no matter the method or the sequencing […] whether it’s a paper-based vignette, a role play, or a low-fidelity simulation, everything, absolutely everything, relies on students having to do clinical reasoning at every pedagogical step. So, it really pushes them to build their clinical reasoning quickly.” (Designer4)

The challenge, as expressed by all stakeholders, was that the methods require sustained engagement and preparation from both students and teachers to ensure their optimal impact on learning. If the level of engagement decreases either from students or teachers, the activity loses some of its substance through reduced participation, thereby lessening its educational impact and potentially hindering the development of students’ CR.

## Discussion

This study aimed to describe how a preclinical CR curriculum aligned with the current recommendations on teaching CR [1] contributes to students’ CR development from multiple perspectives (students, teachers, and designers). The institution’s CR curriculum embedded into the larger undergraduate MD program is based on an explicit definition of CR that encompasses diagnosis and management, knowledge and processes, and perception of the patient as a partner within a particular context. It is anchored in sound theoretical frameworks and models of CR. In its general characteristics, this curriculum as intended seems to be aligned with the current educational recommendations on the ‘what ‘and ‘how ‘of such a curriculum [1]. CR and its domains proposed by Cooper et al. [1] form the consistent matrix of the LU-IW. The focus group results indicate that this is indeed what occurs in practice. The knowledge and skills relevant to understanding, solving, and managing the clinical problems are acquired, combined, organized and applied by students, who progressively engage in CR practice even at this early stage of training.

Effective teaching strategies for CR are well operationalized within each teaching method. Active learner-centred methods, strategies that build understanding, strategies that structure knowledge around problem-specific concepts, retrieval practice, and strategies that allow practice with feedback, are all apparent throughout the LU-IW that we studied. However, all these methods depend on continuous engagement from both students and teachers, which requires strong and ongoing support from the program.

Teachers’ expertise in teaching CR is often reported as a significant barrier for implementation. While clinical preceptors may excel at CR in their practice, coaching students on the basics of CR remains a challenge [1,5,7,18]. In our study, we did not observe any perceptions from the different stakeholders’ groups of insufficient CR teaching skills from teachers. This discrepancy with the literature may be explained by the massive, systematic, and ongoing investment in faculty development, as well as the experience teachers have gained over five years of implementation.

A key characteristic of the CR curriculum, clearly reflected in the LU-IW under analysis, is that it is embedded and deeply imbricated into the UGME program, which is designed, itself, as a fully integrated and spiral curriculum [19]. CR is knowledge-based and there is no evidence that it can be taught efficiently as a separate process. Thus, this full integration of CR with acquisition and organisation of biomedical and clinical knowledge provides consistency and may represent an ideal design. It also yields rationale and support for initiating teaching CR as early as the first year of medical school. However, it entails challenges. First, it practically requires a simultaneous global curriculum renewal to make it happen. In our case, this was feasible due to an ongoing complete program renewal, as was the case for the highly integrated CR curriculum described by Rowat and Suneja [8]. Second, the vertical and horizontal integration requires an interdisciplinary approach, both for students acquiring CR skills as well as for faculty teaching them. It may mean that teachers will sometimes feel outside their comfort zone, and this necessitates appropriate faculty development, healthy collaboration among teachers [20], and high-quality teaching materials. The segmentation and spread over the program of disciplinary content may also make the big picture less obvious for teachers and lead to sparser interactions and potentially more superficial CR coaching with students. On the other hand, students might have to negotiate the depth of their learning so that the disciplinary knowledge they construct at one time has enough internal consistency to be retained and used for CR as well as serve as a foundation to build on, later, when the subject will be revisited.

Our data suggest that students’ cognitive overload, though multifactorial, may revolve at least in part around learning CR. Although some authors have proposed that integrating explicit CR curricula into a program might not require significant additional time [1], the processes of acquiring specific knowledge and skills, integrating them meaningfully, and applying them through explicit CR represent a cumulative challenge for students, albeit one that becomes more accessible with increased familiarity with the educational process. Therefore, educators must remain aware of this challenge and not underestimate the amount of time required by students for integrating CR learning. They must provide continuous support and a tailored structure to meet students’ evolving needs. UGME training alone cannot fully equip students with all the competencies required for CR. This expertise continues to develop throughout residency and into clinical practice.

### Limitations

Our study presents some limitations. This study focuses on a representative segment of one CR curriculum within a single university. Although all LU-IW are designed similarly, there may be unique or subtle variations across the rest of the curriculum that were not fully captured here. This study on curricular issues was conducted with the rigour proposed by Cook et al. [21], and the curriculum was reported according to the recommendations by Hawks et al. [3], to provide insights into the mechanistic understanding of an educational process for CR development relevant beyond our specific institutional context.

## Conclusion

This study provides insights from different stakeholders on the positive contribution to students’ CR development of a preclinical CR curriculum aligned with the current recommendations in the literature. Given the complexity of CR, the design and implementation of such a CR curriculum into a program still represent a challenging task for educators. Students and teachers need appropriate preparation and continued support. Future research could examine a CR curriculum segment over a longer period to better explore the longitudinal nature of the teaching and learning of CR at the undergraduate level. Further research could also examine how these educational approaches ultimately improve students’ CR in practice.

## Additional Files

The additional files for this article can be found as follows:

10.5334/pme.2448.s1 Supplementary File 1. Description of the teaching methods.

10.5334/pme.2448.s2 Supplementary File 2. Interview guide for focus groups.
