# Supplementary material for: Embedding Clinical Reasoning into an Undergraduate Medical Curriculum: A Multi-Stakeholder Perspective
Source: Perspect Med Educ. 2026 Mar 25;15(1):313–21. doi: 10.5334/pme.2448 (PMC13025282; doi:10.5334/pme.2448)
Supplement: Supplementary File 1. — Description of the teaching methods. [file pme-15-1-2448-s1.pdf]

Supplementary file 1 - Description of the teaching methods

Teaching methods and strategies of the LU and IW

The LU includes different teaching methods in which CR is explicitly embedded, along with the number of activities per method: Problem based learning (PBL) (3 activities), Team based learning (TBL) (3 activities), Clinical Skills Communication and Professionalism (CSCP) sessions (4 activities), which includes medical interview (2 activities), physical examination (2 activities) and high-fidelity simulation (1 activity). See Table 1 for details about case format for each teaching method, student's CR tasks, and clinical problems seen. Other learning sessions complete the LU: ECG interpretation; Anatomy and basic echocardiogram; Basic Cardiac Life Support (BCLS).

As is true of the entire curriculum, teaching methods in this five-week section have the following characteristics:
a) Learner-centered, requiring continued active learner participation for knowledge building and understanding.
b) Collaborative: small-groups and teams requiring students' individual preparation and active interactions with peers and teachers.
c) Clinical presentation-centered: starting from clinical presentation to disease rather than the other way around.
d) Clinical cases represent key material for knowledge organisation through the different methods with a high number of cases (total of 46 cases in this LU-IW) and a variety of case formats which evolve according to the stage of learning and CR related tasks expected of students.
e) Through acquiring medical knowledge as well as procedural skills, students are asked concurrently to integrate and progressively apply them for CR about the key clinical presentations. The sequence of methods and content is deliberately organized to support the progression of CR over the course of the LU.

During the IW, the self-explanation and structured reflection activity is a web-based individual learning activity in which students use these strategies to solve and initiate management for three complex cases. The small group standardized patient (SP) session involves students interviewing and examining an SP within a clinical scenario that unfolds in successive time points. Detailed descriptions of the IW methods are presented in Table 2. During the session, students receive feedback from the SP, from their peers, and from the teacher on various aspects, including CR, communication skills, professionalism, and patient-centered management. During the IW, students concurrently prepare for periodic summative assessment.

Through all these teaching methods, teachers function as facilitators for students' knowledge building and understanding, acting concurrently as explicit coaches and models for CR. The teacher's guide is constructed with emphasis on gathering information, generating diagnostic possibilities, problem representation, diagnostic

justification, revision prompted by new data or unexpected clinical course, communication, professionalism, patient-centered management, environmental and social constraints. These processes figure explicitly in both the students' and the teachers' manuals across the range of clinical scenarios. They are also reflected repeatedly and consistently in the questions that the students are asked to address.

Table 1. Description of case format, students' clinical reasoning tasks and clinical problems seen in each of the LU teaching method

Teaching methods	Case formats	Students' CR related tasks	Clinical problems		
			**Palpitations-syncope Diagnosis**	**Thoracic pain Diagnosis**	**Shortness of breath Diagnosis**
PBL (3)<br>Small group	- Whole case<br>- Complete infos from symptoms to planning follow up<br>- Prototypical<br>- Written case	Understand and explain biomedical mechanisms and clinical knowledge	1 case:<br>- Atrial fibrillation, rapid response rate	1 case:<br>- Coronary artery disease (CAD)	1 case:<br>- Chronic obstructive pulmonary disease (COPD) exacerbation
TBL phase III (3)<br>Small group	- Serial cue case<br>- Complete infos from history to management<br>- Prototypical<br>- Written case	Diagnostic reasoning and initiation of management prompted by questions	4 cases:<br>- Syncope: Complete atrioventricular block<br>- Thoracic pain: aortic stenosis<br>- Orthostatic syncope<br>- Neurocardiogenic syncope	4 cases:<br>- Pulmonary embolism<br>- Gastric reflux<br>- Pericarditis<br>- Anxiety-thoracic zona	4 cases:<br>- CAD - Heart failure Reduced ejection fraction<br>- Professional asthma<br>- Pulmonary fibrosis<br>- Heart failure Preserved ejection fraction and Hypertension
Clinical Skills Communication and Professionalism (CSCP)<br>Small group					
Medical interview Session (2)					
1 - Role-playing (time in, time out)<br>Small group interview with the teacher as the patient	Role-play: history only	- Practice interview and hypotheses-driven data collection<br>- problem representation and differential diagnosis	1 case:<br>- Atrial fibrillation and hyperthyroidism	1 case:<br>- Anxiety post myocardial infarction	1 case:<br>- Cardiac failure in COPD patient

Teaching methods	Case formats	Students' CR related tasks	Clinical problems		
			**Palpitations-syncope Diagnosis**	**Thoracic pain Diagnosis**	**Shortness of breath Diagnosis**
2- Role-play by students, in student triads	Role-play: history only	- Practice interview and hypotheses-driven data collection<br>- Provide feedback	3 cases:<br>- Palpitations: Paroxysmal supraventricular tachycardia<br>- Syncope induced by nitro in CAD<br>- Palpitation-syncope: CAD and Ventricular tachycardia	3 cases:<br>- Viral pericarditis<br>- Unstable angina<br>- Post partum Pulmonary embolism	3 cases:<br>- Cardiac failure<br>- Allergic asthma<br>- Sarcoidosis
Physical examination (lungs-heart-vascular) (1) Demonstration by teacher followed by individual practice in triads	- Written case: history only	- Observe procedures<br>- Practice procedures and hypotheses-driven data collection<br>- Provide feedback	3 cases:<br>- Thoracic pain and shortness of breath: Acute coronary syndrome and cardiac failure<br>- Shortness of breath and acute chest pain: Pulmonary embolism<br>- Shortness of breath and cough: Pneumonia		
Cardiac auscultation workshop (1)	- Written cases with audio recording of abnormal findings	- Describe abnormal findings<br>- Interpret and provide probable diagnosis	10 cases:<br>- Pericarditis<br>- Mitral insufficiency: endocarditis<br>- Aortic insufficiency: Marphan<br>- Aortic stenosis<br>- Hypertrophic cardiomyopathy<br>- 3rd sound: cardiac failure<br>- Mitral insufficiency chronic<br>- Mitral stenosis<br>- congenital heart disease<br>- 4th sound: ventricular hypertrophy		

Teaching methods	Case formats	Students' CR related tasks	Clinical problems		
			Palpitations-syncope Diagnosis	Thoracic pain Diagnosis	Shortness of breath Diagnosis
Workshop Simulation in the lab (1)	- High fidelity simulated case	- Urgent team intervention<br>- Basic Cardiac Life Support<br>- Team collaboration and communication	2 cases:<br>- Cardiac arrest in the community<br>- Cardiac arrest in outpatient clinic		

Table 2. Description of case format, students' clinical reasoning tasks and clinical problems seen in each of the IW teaching method

Teaching methods	Case formats	Students' CR related tasks	Clinical problems (of two previous LU: Hematuria, Oedema, Renal failure, Palpitation-syncope, Thoracic pain, Shortness of breath)
Self-explanation-Structured reflection activity (1)<br>Individual online learning	- Whole case<br>- Less typical<br>- Undifferentiated<br>- Written cases	Diagnostic reasoning:<br>- Self-explain while solving the case<br>- Complete a structured reflection grid with the differential diagnosis<br>- Initial laboratory testing and management	3 cases:<br>- Acute shortness of breath: asthma<br>- Acute renal failure: acute urinary retention<br>- Syncope: sick sinus syndrome
Standardized patient session (1)<br>Small group	- Complete clinical scenario role-played with a trained standardized patient	- Early hypotheses generation<br>- 3 successive students: hypotheses-driven interview and physical exam (observation by other students and debrief after each student)<br>- problem representation<br>- differential diagnosis, - EKG and lung X-rays interpretation	1 case: acute myocardial infarction with pulmonary oedema
