# Supplementary material for: Embedding Clinical Reasoning into an Undergraduate Medical Curriculum: A Multi-Stakeholder Perspective
Source: Perspect Med Educ. 2026 Mar 25;15(1):313–21. doi: 10.5334/pme.2448 (PMC13025282; doi:10.5334/pme.2448)
Supplement: Supplementary File 2. — Interview guide for focus groups. [file pme-15-1-2448-s2.pdf]

Supplementary file 2 - Interview guide for focus groups*

* The interview guide was translated from French. For each question, we provide the different versions adapted for each group.

Groups	Main questions	Prompts
Designers, teachers-LU and teachers-IW	1. In general, in your opinion, how does the curricular structure — learning unit followed by an integration week — contribute to the development of students' clinical reasoning?	1a) Is there anything that comes to mind regarding the methods that support the development of students' clinical reasoning?<br><br>1b) Is there anything that comes to mind regarding the content that is taught — whether in terms of knowledge or skills — that, in your view, supports the development of students' clinical reasoning?<br><br>1c) Is there anything that comes to mind regarding the sequence — the arrangement of content and methods within a learning unit — how it is organized in a certain order?
Students	1. In general, in your opinion, how does the curricular structure — learning unit followed by an integration week — contribute to the development of your clinical reasoning?	1a) Is there anything that comes to mind regarding the methods that support the development of your clinical reasoning?<br>1b) Is there anything that comes to mind regarding the content that is taught — whether in terms of knowledge or skills — that, in your view, supports the development of your clinical reasoning?<br>1c) Is there anything that comes to mind regarding the sequence — the arrangement of content and methods within a learning unit — how it is organized in a certain order?
Designers, teachers-LU and teachers-IW	2.1. What are the strengths of this curricular structure — learning unit followed by an integration week — (or within this structure: content, methods, and sequencing) for the	

	development of students' clinical reasoning?	
Students	2.1. What are the strengths of this curricular structure — learning unit followed by an integration week — (or within this structure: content, methods, and sequencing) for the development of your clinical reasoning?	Prompt: Tell me about the repetition of the learning units — how is this a strength that supports the development of your clinical reasoning?
Designers, teachers-LU and teachers-IW	2.2. Now, looking at it from the other side, what are the limitations of this curricular structure — learning unit followed by an integration week — (content, methods, sequencing) for the development of students' clinical reasoning?	2.2a) Are there any limitations or aspects that do not work well regarding the content?<br><br>2.2b) Are there any limitations regarding the methods?
Students	2.2. Now, looking at it from the other side, what are the limitations of this curricular structure — learning unit followed by an integration week — (content, methods, sequencing) for the development of your clinical reasoning?	2.2c) Are there any limitations regarding the sequence?
Students	3. How accessible and understandable was this structure at the beginning? What about the complexity and cognitive load at first — the layout of the material, the learning objectives?	
All groups	Do you have anything else you would like to add about the elements we discussed, or anything we may have missed?	
